# Sub-Micromolar Inhibition of SARS-CoV-2 3CLpro by Natural Compounds

**DOI:** 10.3390/ph14090892

**Published:** 2021-09-01

**Authors:** Bruno Rizzuti, Laura Ceballos-Laita, David Ortega-Alarcon, Ana Jimenez-Alesanco, Sonia Vega, Fedora Grande, Filomena Conforti, Olga Abian, Adrian Velazquez-Campoy

**Affiliations:** 1 CNR-NANOTEC, SS Rende (CS), Department of Physics, University of Calabria, 87036 Rende, Italy; 2 Institute for Biocomputation and Physics of Complex Systems (BIFI), Joint Units IQFR-CSIC-BIFI, and GBsC-CSIC-BIFI, Universidad de Zaragoza, 50018 Zaragoza, Spain; ceballos.laita@gmail.com (L.C.-L.); dortega@bifi.es (D.O.-A.); ajimenez@bifi.es (A.J.-A.); svega@bifi.es (S.V.); 3 Instituto de Investigación Sanitaria de Aragón (IIS Aragon), 50009 Zaragoza, Spain; 4 Departamento de Bioquímica y Biología Molecular y Celular, Universidad de Zaragoza, 50009 Zaragoza, Spain; 5 Department of Pharmacy, Health and Nutritional Sciences, University of Calabria, 87036 Rende, Italy; fedora.grande@unical.it (F.G.); filomena.conforti@unical.it (F.C.); 6 Instituto Aragonés de Ciencias de la Salud (IACS), 50009 Zaragoza, Spain; 7 Centro de Investigación Biomédica en Red en el Área Temática de Enfermedades Hepáticas Digestivas (CIBERehd), 28029 Madrid, Spain; 8 Fundación ARAID, Gobierno de Aragón, 50018 Zaragoza, Spain

**Keywords:** SARS-CoV-2, main protease, eugenol, drug selection, enzyme inhibitors, antivirals, spectroscopy, molecular modeling

## Abstract

Inhibiting the main protease 3CLpro is the most common strategy in the search for antiviral drugs to fight the infection from SARS-CoV-2. We report that the natural compound eugenol is able to hamper in vitro the enzymatic activity of 3CLpro, the SARS-CoV-2 main protease, with an inhibition constant in the sub-micromolar range (K_i_ = 0.81 μM). Two phenylpropene analogs were also tested: the same effect was observed for estragole with a lower potency (K_i_ = 4.1 μM), whereas anethole was less active. The binding efficiency index of these compounds is remarkably favorable due also to their small molecular mass (MW < 165 Da). We envision that nanomolar inhibition of 3CLpro is widely accessible within the chemical space of simple natural compounds.

## 1. Introduction

The COVID-19 pandemic caused by the severe acute respiratory syndrome coronavirus 2 (SARS-CoV-2) is having a strong impact on the social and economic conditions worldwide, as well as on the scientific community. Highly collaborative efforts have been started to tackle the emergency, with the aim of improving the detection of infections, tracing the occurrence of potentially contagious contacts, adjusting the pre-existing medical therapies, developing vaccines for prevention and monoclonal antibodies for early treatment, and identifying new drugs against this viral infection. Unfortunately, discovering specific antiviral compounds against SARS-CoV-2 is still demanding. Currently, only the broad-spectrum drug Remdesivir has been approved [1], in spite of its relatively low activity.

The coronavirus genome contains two overlapping open reading frames (ORF1a and ORF1b) encoding polyproteins pp1a and pp1ab. These polyproteins are processed by a 3C-like protease (3CLpro or main protease, Mpro), responsible for cleaving at eleven sites, and a papain-like protease (PLpro), responsible for cleaving at three sites. Both 3CLpro and PLpro are essential for viral replication, making them attractive targets for drug development. Most of the efforts to develop an antiviral drug specific for SARS-CoV-2 aim at inhibiting the main protease 3CLpro, due to its key role in the virus replication [2]. 3CLpro is a cysteine protease with an active site located in the cleft between two N-terminal domains and containing a catalytic Cys-His dyad.

A plethora of molecules have been proposed in the literature as potentially active against this pharmacological target, especially by using computational predictions [3,4,5]. Some of these have been confirmed to bind 3CLpro by experimental methods, and have shown promising inhibitory effects. Among the molecules investigated, natural compounds have a prominent role [6] due to their large diffusion, high variety of molecular features, and wide spectrum of effects in cells. Molecules with confirmed in vitro inhibitory activity against 3CLpro include baicalein [7,8], quercetin [9], rutin [10], epigallocatechin-3-gallate [11], and myricetin [12,13].

We have contributed to this community effort by using an experimental pipeline for drug screening formerly employed to identify inhibitors against other protein targets [14,15,16,17]. We have successfully redirected this platform to tackle 3CLpro from SARS-CoV-2. In particular, we have identified quercetin as a low micromolar inhibitor (K_i_ = 7.4 μM), a high potency, especially considering its small molecular mass (MW = 302.2 Da), which has a favorable outcome in terms of a high binding efficiency index (BEI = pK_i_/MW) [9]. Subsequently, we demonstrated that a natural glycoside form of quercetin, rutin, has a comparable potency against 3CLpro (K_i_ = 11 μM) [10]. Thus, their common scaffold can be considered promising for further optimization. A seleno-functionalized quercetin analog was later proved to inhibit SARS-CoV-2CoV-2 replication in infected cells at non-toxic concentration, with an IC_50_ value of 8 μM [18].

**Figure 1:**
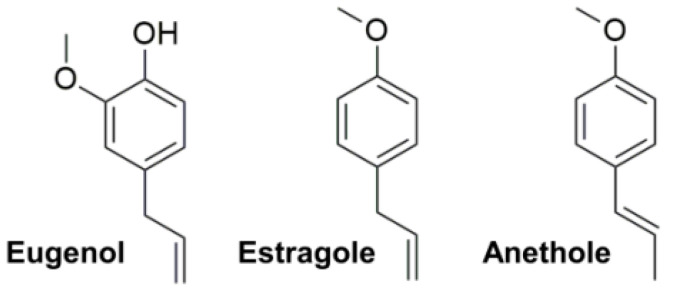
Chemical structure of eugenol, estragole, and anethole.

In our search for other scaffolds with favorable potency to be used as 3CLpro inhibitors, and among hundreds of compounds tested so far, we have also considered three well-known phenylpropenes [19]: eugenol, estragole, and its isomer anethole (see Figure 1).

These terpenes are volatile substances commonly found in spices and aromatic herbs including clove, basil, cinnamon, pepper, fennel, and anise [20], and are generally considered safe for human use [21]. They have a small molecular mass (eugenol has MW = 164.2 Da, and the other two compounds have MW = 148.2 Da), well within the conventional limit (MW < 300 Da) for considering them as chemical fragments. The compounds were tested against recombinant 3CLpro, which was expressed and purified as previously described [9]. In this communication we report their notable inhibitory activity, provide some suggestions on using their scaffold in a lead compound development and, more importantly, set a new landmark reference encouraging further research on the use of natural products (or their derivatives) as antivirals against SARS-CoV-2.

## 2. Results and Discussion

A combination of experimental and computational techniques were used to characterize the interaction of eugenol, estragole, and anethole with the main protease 3CLpro, with particular regard to their inhibitory properties.

**Figure 2:**
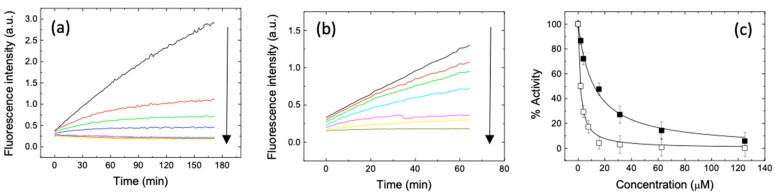
(**a**) Fluorescence emission of the substrate (concentration 20 μM) in the presence of 3CLpro (0.2 μM) as a function of time, at varying amounts (0–125 μM) of (**a**) eugenol and (**b**) estragole. The initial slope of the curves quantifies the enzymatic protein activity. The arrows indicate the increase in inhibitor concentration. (**c**) Inhibition curve for eugenol (open squares) and estragole (closed squares). The continuous line is from a non-linear least squares regression fit, and provides the inhibition constant (K_i_ = 0.81 and 4.1 μM for eugenol and estragole, respectively). The different colors in (**a**,**b**) correspond to the different concentrations shown in (**c**): 0 (black), 1.95 (red), 3.91 (green), 7.81 (blue), 15.63 (cyan), 31.25 (magenta), 62.5 (yellow), and 125 μM (brown).

Figure 2 shows the data obtained for eugenol and estragole, by using the same experimental setup and conditions already reported [9,10,18]. The catalytic activity of 3CLpro was monitored in vitro by using a Förster resonance energy transfer (FRET) continuous assay, in the presence of the substrate (Dabcyl)KTSAVLQSGFRKME(Edans)-NH2. The enzyme activity was quantitated as the initial rate (slope) for each substrate FRET emission curve, which varied as a function of the compound concentration. By assuming a simple inhibition process, and through a non-linear regression applying a model that considers inhibitor depletion due to the binding, this yielded an apparent inhibition constant K_i,app_ of 1.7 and 12 μM for eugenol and estragole, respectively. In contrast, anethole was less potent (K_i,app_ of 25 μM, data not shown).

**Figure 3:**
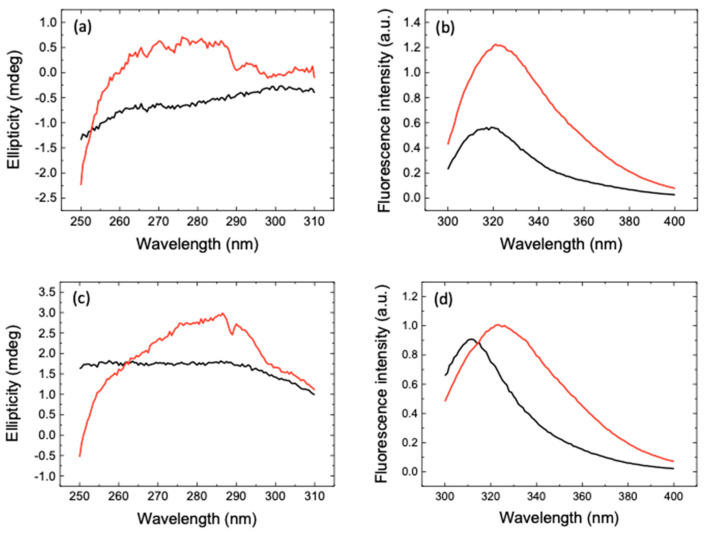
(**a**) Near-UV CD and (**b**) fluorescence emission spectra of the 3CLpro-eugenol complex (black lines) at enzyme and inhibitor concentrations of 10 and 100 μM, respectively. The addition of individual spectra of 3CLpro and eugenol is also shown (red lines). (**c**) Near-UV CD and (**d**) fluorescence emission spectra of the 3CLpro-estragole complex (black lines) at enzyme and inhibitor concentrations of 10 and 100 μM, respectively. The addition of individual spectra of 3CLpro and estragole is also shown (red lines). The non-equivalence between the spectrum of the complex and the addition of the individual spectra of the free species provides direct evidence for the enzyme–inhibitor interaction.

A further analysis of the data, by taking into account the substrate concentration and the competitive inhibition [9,10], led to an estimation of the intrinsic inhibition constant Ki of 0.60 and 4.3 μM for eugenol and estragole, respectively, the two most active compounds (see again Figure 2). Furthermore, to confirm target engagement for each compound, we used near-UV circular dichroism (CD) and fluorescence emission. This region of the CD spectrum is particularly sensitive to subtle changes in the protein structure and in the environment of aromatic residues, which include the key histidine in the catalytic dyad of 3CLpro (His41/Cys145) [22]. As shown in Figure 3, the CD and fluorescence spectra confirmed that eugenol binds to 3CLpro. Similar results were obtained for estragole (Figure 3).

**Figure 4:**
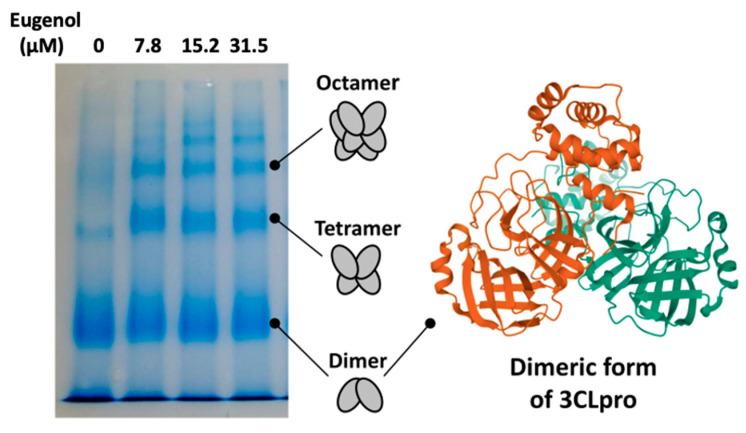
Native polyacrylamide gel electrophoresis (PAGE) showing the effect of eugenol on 3CLpro quaternary structure. The lanes correspond to eugenol concentrations of 0, 7.8, 15.2, and 31.5 μM (from left to right).

3CLpro shows a complex conformational/functional landscape. Its active conformation is homodimeric, whereas the monomeric form is inactive. However, the presence of other high-order oligomers (e.g., tetramers and octamers) with considerable hydrolytic activity has been reported [9,23]. It is then possible that ligands binding to 3CLpro may modulate its conformational equilibrium, and even act as inhibitors by shifting it towards the inactive monomeric state [24]. Interestingly, we found that under our experimental conditions 3CLpro populated mainly dimers, with a minor fraction of larger oligomers, but the interaction with eugenol resulted in a considerable increase in high-order quaternary structures, as shown by electrophoresis (Figure 4). This observation, combined with 3CLpro engagement demonstrated by the CD and fluorescence data (Figure 3), suggests that eugenol exerts its inhibition activity as a direct action on the protein catalytic site and, in turn, it has an allosteric effect that promotes 3CLpro oligomerization.

In the absence of structural data to confirm the exact location of the ligand bound to 3CLpro, we used computational techniques to gain insight into the anchoring mode, as previously described [9,10,18]. The results of docking simulations performed with AutoDock Vina [25] predicted the binding into the 3CLpro catalytic site with affinity at least in the micromolar range (binding energy < −5.0 kcal/mol). Random errors on the docking energies were very low due to the high exhaustiveness used (≤0.1 kcal/mol), and, therefore, uncertainties were essentially due to the systematic error of the scoring function of AutoDock Vina [25]. Remarkably, the binding score obtained in the docking to unliganded 3CLpro structures present in the Protein Data Bank (PDB) repository (such as entries 6Y2E [22] and 7JUN [26] obtained by, respectively, X-ray and neutron crystallography) was significantly less favorable than the value observed in vitro. This observation could explain why neither eugenol nor estragole were reported in previous large-scale computational screening of flavonoids [27,28]—at variance with other natural compounds later confirmed to inhibit 3CLpro.

**Figure 5:**
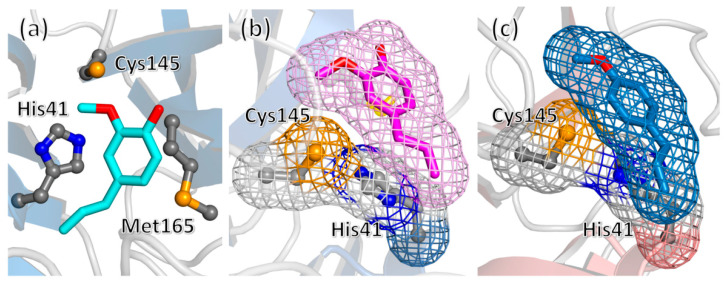
(**a**) Docking pose (binding energy of –5.7 kcal/mol) of eugenol in the active site of 3CLpro (protein extracted from PDB structure 7DPU), interacting with the protein side chains of the catalytic dyad His41/Cys145 and with residue Met165. (**b**) Similar binding mode obtained for another 3CLpro structure (entry 6YNQ), shown in a different orientation to highlight the interaction of the propanoid tail with residue His41. (**c**) One of the binding modes of estragole to unliganded 3CLpro (protein from PDB entry 6Y2E).

The result obtained in the docking experiments could be affected, at least in part, by the limitation of this technique due to the fact that the protein structure is considered rigid during the simulation, combined with the structural plasticity of the 3CLpro site in hosting chemical fragments [29]. Thus, we extended our molecular docking experiment to 3CLpro structures extracted from protein–ligand complexes. As shown in Figure 5, a consensus was obtained on a binding mode of eugenol consisting in the compound closely interacting with its propanoid tail with the catalytic residue His41, possibly hindering, in this way, its catalytic activity. The binding affinity was <–5.5 kcal/mol, and could be further improved up to the threshold of –6.0 kcal/mol by employing molecular dynamics (MD) simulations to adjust the accommodation of the ligand (following a protocol already used for investigating the 3CLpro/rutin complex [10]). A comparable value was obtained for the most favorable binding mode of estragole, although its binding energy was slightly lower.

Due to the extensive MD sampling performed, compared to the relatively short timescale necessary to equilibrate the position of our small ligands (below 1 ns), in this case, the uncertainties on the binding affinity could be assumed to be essentially dictated by the systematic error on the force field used. Within these limitations, the simulation results are consistent with a plausible association of both eugenol and estragole within the binding site of 3CLpro.

The binding affinities and inhibitory effects found for the phenylpropenes here investigated, and especially for eugenol, are noteworthy for a number of reasons. Foremost, and to the best of our knowledge, this is the first report of a natural compound that inhibits 3CLpro with a potency in the (high) nanomolar range. Whereas other compounds with sub-micromolar potency have been found in the last few months, they have higher molecular weight (thus, lower BEI) and have been designed by brute-force approaches to optimize the binding to 3CLpro [3,5]. Importantly, although those synthetic compounds are modeled to avoid potentially inappropriate pharmacokinetic properties, many of them interact covalently and their toxicity and metabolic profile is yet unknown. In contrast, our natural compounds have low toxicity (LD_50_ value for eugenol is >1930 mg kg^–1^ in rodents, and even higher for the other phenylpropenes) [30]. In particular, eugenol is already considered a safe food additive, and it has been widely used as an oral disinfectant in dentistry for more than a century [31]. Furthermore, its antiviral properties have already been reported in a number of cases, for instance, it has been demonstrated to inhibit *Herpes simplex* virus in vitro [32].

Other reasons for an interest in the compounds here reported are due to their molecular properties. The fact that eugenol and estragole are both highly active to inhibit 3CLpro strongly suggests that the phenylpropanoid scaffold is responsible for this feature. Furthermore, the decrease in the inhibitory efficiency in the comparison between estragole and anethole, which differ solely by the position of a double bond, indicates that the flexibility due to dihedral angle rotations in the –CH_2_–CH=CH_2_ moiety is a molecular feature that favors the bioactivity of both eugenol and estragole. All of these findings may provide useful indications for the use of eugenol as a lead compound, with the aim to attempt an optimization of its molecular scaffold against 3CLpro. This process may follow the same route that has led to improvements of the non-covalent binding and antiviral activity of quercetin, through a synthetic functionalization [18]; or to the rational selection of pyrogallol-containing natural products starting from myricetin [33], which covalently bind the protein catalytic residue Cys145.

## 3. Materials and Methods

### 3.1. Chemical Compounds

For our experiments, eugenol and estragole were purchased from Alfa Aesar—Thermo Fisher Scientific (Karlsruhe, Germany) and anethole from Sigma-Aldrich (Milan, Italy), all in liquid form and with purity ≥ 98%, and they were dissolved in assay buffer (Tris 50 mM, pH 7).

### 3.2. CLpro Expression and Purification

SARS-CoV-2 3CLpro (ORF1ab polyprotein residues 3264-3569, GenBank code: MN908947.3) was expressed using a His-tagged construct in a pET22b plasmid transformed into BL21 (DE3) Gold *E. coli* strain, as reported previously [9,10,18]. Briefly, after induction of expression with isopropyl 1-thio-β-D-galactopyranoside in cells grown in LB/ampicillin media, the soluble protein extract, obtained by sonication rupture, was purified using metal affinity chromatography (cobalt HiTrap TALON column, GE-Healthcare Life Sciences, Barcelona, Spain). After dialysis in storage buffer (sodium phosphate 50 mM, pH 7, sodium chloride 150 mM), an extinction coefficient of 32,890 M^−1^ cm^−1^ at 280 nm was employed for protein concentration quantification.

### 3.3. CLpro Catalytic Activity

In vitro catalytic activity of 3CLpro was monitored using a Förster resonance energy transfer (FRET) continuous assay with the substrate (Dabcyl)KTSAVLQSGFRKME(Edans)-NH2 (Biosyntan GmbH) [22]. This substrate contains the nsp4/nsp5 cleavage sequence, GVLQ↓SG. The enzymatic reaction was initiated by adding substrate at 20 μM to the enzyme at 0.2 μM in a final volume of 100 μL, in assay buffer: sodium phosphate 50 mM, NaCl 150 mM, pH 7. The initial rate was determined in a FluoDia T70 microplate reader (Photon Technology International, Birmingham, NJ, USA) for 20 min (excitation wavelength, 380 nm; emission wavelength, 500 nm; both wavelengths are the closest possible to those indicated by the substrate manufacturer). The readout was the fluorescence intensity increase due to diminished FRET in the substrate as it was hydrolyzed by 3CLpro. Initial rate was estimated as the initial slope in the fluorescence intensity as a function of time. The Michaelis–Menten constant, *K_m_*, and the catalytic rate constant or turnover number, *k*_cat_, were estimated previously (*K*_m_ = 11 μM and *k*_cat_ = 0.040 s^−1^) [14].

### 3.4. Inhibition Assay

The inhibition constant for each compound was estimated by measuring the enzyme activity as a function of compound concentration: enzyme at 0.2 µM final concentration was incubated with compound at a concentration 0–125 µM for (at least) 30 min, initiating the reaction by adding substrate at 20 µM final concentration [9,10,18], in assay buffer: sodium phosphate 50 mM, NaCl 150 mM, pH 7. The initial slope of the substrate fluorescence emission time curve was processed to calculate the percentage of inhibition at each compound concentration. The initial rate was estimated as the initial slope in the fluorescence intensity as a function of time. The initial slope ratios provided the percentage of activity or the percentage of inhibition. Non-linear regression analysis employing a simple inhibition model (considering inhibitor depletion due to enzyme binding) allowed us to estimate the apparent inhibition constant for each compound [9,10]. Assuming competitive inhibition, the intrinsic (i.e., substrate concentration-independent) inhibition constants were determined.

Different controls were included in all activity measurements: substrate with no protease, no substrate with protease, and substrate with protease. As expected, no activity was observed in the first and second cases, and maximal activity was observed in the third case. In addition, from our work, several compounds inhibiting SARS-CoV-2 3CLpro have already been identified and reported (e.g., quercetin [9] and rutin [10]), and they were used as positive controls for 3CLpro inhibition. Experiments were performed in duplicates, at least. Non-active compounds exhibited maximal protease activity (around 100% activity) at any concentration.

### 3.5. Spectroscopy: Circular Dichroism and Emission Fluorescence

Although far-UV circular dichroism is easier to interpret in terms of structural features of the protein, near-UV circular dichroism is more sensitive to slight alterations in the microenvironment of the protein aromatic residues and, therefore, a better reporter of the potential protein–compound interaction. Near-UV circular dichroism spectra were recorded in a Chirascan spectropolarimeter (Applied Photophysics, Leatherhead, UK) at 25 °C, in a 1 cm path length cuvette and employing a protein concentration of 10 μM and a compound concentration of 100 μΜ. Only raw ellipticity (reported in mdeg) was considered because only the influence of the compounds on the protein target was assessed. Spectroscopic measurements were made in sodium phosphate 50 mM, pH 7.

Fluorescence spectroscopy was employed for probing the solvent-exposure of the three tryptophan residues of 3CLpro as a reporter for the potential protein–compound interaction using a Cary Eclipse spectrofluorimeter (Agilent, Santa Clara, CA, USA). Emission spectra were recorded between 300 and 400 nm (excitation at 290 nm) at 25 °C, in a 1 cm path length cuvette and employing a protein concentration of 10 μM and a compound concentration of 100 μΜ. Spectroscopy experiments were performed in duplicates, at least.

### 3.6. PAGE Native Electrophoresis

The potential modulation of the quaternary assembly of 3CLpro by the compounds was assessed through PAGE native electrophoresis. A fixed protein concentration (7 μM) was incubated (overnight at 4 °C and 1 h at room temperature before the experiment) with increasing concentrations of compounds (0, 7.8, 15.2, and 31.5 μM) and run through a 12% acrylamide gel. After staining with Coomassie Blue, the different oligomeric forms could be observed.

### 3.7. Molecular Docking

The association between 3CLpro and the compounds tested was assessed by using the docking engine AutoDock Vina 1.1.2 [25] following the same protocol already described for modeling the binding with quercetin [9] and its analogs [10,18]. The protein was extracted from PDB structures, either in unliganded form [22,26] or complexed with a variety of small chemical fragments [29]. A blind search was carried out at high exhaustiveness, 16 times larger than the default value [34], resulting in an exponentially larger probability of finding the most favorable binding mode. The whole protein was considered as rigid and with full flexibility for the ligands around their rotatable dihedral angles.

### 3.8. Molecular Dynamics

The protein–ligand complexes obtained through docking simulations were refined by MD in explicit water, performed using the GROMACS package in combination with the force field Amber ff99SB-ILDN and GAFF, with the same procedure formerly reported for the 3CLpro-rutin complex [10]. After preparation through energy minimization, annealing, and equilibration, the production runs were carried out at constant pressure and temperature for 10 ns. Integration time was 2 fs (with constrained bond distance for non-hydrogen atoms), sampling was every 1 ps, and all other simulation conditions were as previously reported [35].

## 4. Conclusions

The overall results presented in this communication, obtained by using a combination of experimental and computational techniques already successfully employed to find inhibitors for the main protease 3CLpro from SARS-CoV-2, indicate that eugenol is a potent inhibitor of this protein that is active at high nanomolar concentrations. This property is in common with estragole (albeit with lower potency), and less pronounced for anethole. These findings provide indications that natural substances may be used directly against COVID-19, under appropriate conditions or formulations. The phenylpropanoid scaffold could be used in rational design endeavors as a chemical fragment for drugs aimed at inhibiting 3CLpro. However, as happens with any drug to be developed, some potential limitations can be foreseen, stemming mainly from the pharmacokinetics of these compounds once administered (i.e., absorption–distribution–metabolism–excretion properties, ADME), which would determine their bioavailability and their effective concentration for inhibiting viral replication at the proper intracellular location. Thus, the appropriate formulation and administration routes will be key for therapeutic success. Regardless of this, the results reported in this work indicate that nanomolar inhibition of 3CLpro by simple bioactive molecules is within reach, and our findings may represent a milestone towards the discovery of other natural compounds with more favorable properties.

## Data Availability

Data is contained within the article.
